# GC/MS Analysis of Fatty Acids on *Pliek U* Oil and Its Pharmacological Study by Molecular Docking to Filaggrin as a Drug Candidate in Atopic Dermatitis Treatment

**DOI:** 10.1155/2019/8605743

**Published:** 2019-11-03

**Authors:** Nanda Earlia, Muslem, Rivansyah Suhendra, Mohamad Amin, C. R. S. Prakoeswa, Khairan, Rinaldi Idroes

**Affiliations:** ^1^Graduate School of Mathematics and Applied Sciences, Universitas Syiah Kuala, Banda Aceh 23111, Indonesia; ^2^School of Medicine, Universitas Syiah Kuala, Banda Aceh 23111, Indonesia; ^3^Department of Chemistry, Faculty of Mathematics and Natural Sciences, Universitas Syiah Kuala, Banda Aceh 23111, Indonesia; ^4^Department of Informatics, Faculty of Mathematics and Natural Sciences, Universitas Syiah Kuala, Banda Aceh 23111, Indonesia; ^5^Department of Biology, Faculty of Mathematics and Natural Sciences, Universitas Negeri Malang, Malang 65145, Indonesia; ^6^Faculty of Medicine, Universitas Airlangga—Dr Soetomo, General Academic Hospital, Surabaya 60132, Indonesia; ^7^Department of Pharmacy, Faculty of Mathematics and Natural Sciences, Universitas Syiah Kuala, Banda Aceh 23111, Indonesia

## Abstract

Analysis of fatty acid contents and pharmacological properties of *Pliek U* oil was performed. Fatty acids were analyzed by gas chromatography-mass spectrometry (GC-MS), and pharmacological properties based on its potential on filament-aggregating protein (filaggrin) were studied with bioinformatics approach by the reverse docking technique using palmitic acid as a control compound. Two *Pliek U* extracts, namely, *Pliek U* oil (PUO) and ethanolic *Pliek U* oil extract (EPUOE), were prepared. The GC-MS results revealed that lauric acid, myristic acid, palmitic acid, and oleic acid are the predominant fatty acids, with lauric acid being the abundant one in all *Pliek U* oil extracts. The reverse docking technique results showed that oleic acid had the most stable interaction to filaggrin with the lowest binding affinity (−6.1 kcal/mol). Oleic acid and palmitic acid have one same side binding to filaggrin on amino acid LEU D75. These findings indicated that oleic acid has the best potential to be used as a drug candidate in atopic dermatitis treatment.

## 1. Introduction

Skin has two main functions; it acts as an external barrier and provides protection to the body. Skin hinders excessive water loss from the tissue and at the same time protects the body against foreign pathogens. The skin layer that acts as the protection barrier is the widely bound lipid-protein solid matrix called stratum corneum. The primary protein of the lipid-protein matrix is filaggrin [1], while the lipid consisted of ceramides, fatty acids, and cholesterol [2].

Structural damages to the lipid-protein matrix may result in major disfunction of the skin barrier-protection system. Studies on atopic dermatitis reveal damages to filaggrin [1, 3, 4] and deficiency of free fatty acids in the lipid structure as the main causes of diseases [2]. Deficiency of fatty acids in the lipid structure leads to the reduction of skin humidity that causes dry skin. This further causes itching that triggers mechanical damage of the skin barrier-protection system [5, 6].

One of the treatments used to cure atopic dermatitis is applying the moisturizing cream. The moisturizer works to soothe dryness and itching as well as to recover the damaged barrier-protection system. Fatty acids are the main components of moisturizing cream due to their humectant and occlusive effects. Fatty acids also offer pharmacological benefits to repair skin barrier-protection and to accelerate wound healing through rapid epithelization [7–9].

Fatty acids can be found in animal fats and vegetable oils that can be extracted from fruits, seeds, and nuts [10]. Coconut meat has a considerable amount of fatty acids in it. In addition, coconut has been notably identified as a commodity of tropical countries. In Indonesia, especially in Aceh province, coconut is harvested by local farmers, and its meat is traditionally processed through fermentation to produce coconut oil (commonly known as *Pliek U* oil) [7]. *Pliek U* oil is known for its rich fatty acid content, thus allowing this fermented product to be not only treated as a food product but also as a medical potential. Further development of this product, especially as a medical potential, can economically help the local farmers.

However, further studies on the fatty acid content of coconut meat products (such as coconut oil) are required. This is due to the fact that the fatty acid content can be varied depending on the geographical condition of the cultivation, the varieties [11], and the harvesting time [12]. Therefore, it is important to conduct studies that determine the predominant fatty acid that can provide a reference for the development of medical potential of coconut meat products.

Studies on medicinal compound activities against certain proteins generally are done by spectroscopy approaches such as UV-Vis absorption [13], sensor [14], laser, and electrometric methods [15, 16], but today it is carried out predominantly by computational methods, either virtually or *in silico* [17, 18]. Virtual screening with molecular docking is one of the computational methods used for the said purpose. Molecular docking studies are carried out on the structural basis of the screened-potential medicinal compound against the complementary macromolecular binding sides with its protein (structure orientation and electrostatic interaction) [19]. A fatty acid that has the most suitable and stable structural orientation to electrostatically interact with filaggrin protein is then suggested as the highly effective medicinal compound.

In this research, the fatty acids of *Pliek U* oil were analyzed with gas chromatography-mass spectrometry (GC-MS) and further analyzed with the reverse docking technique to evaluate its potential medicinal activities.

## 2. Materials and Methods

### 2.1. Sample Extraction

Coconut fruit samples were obtained from the *Pliek U* raw material supplier in Matang subdistrict, Bireuen district, Aceh province. The samples were prepared to produce *Pliek U* oil (PUO) and ethanolic *Pliek U* oil extract (EPUOE).

The coconut fruits were cleaved and the water was drained off. The fruits were left to dry for 5 days. The meat was grated and placed in a sealed container. The meat was left for another 5 days at room temperature (29–36°C) and then fermented. The fermented coconut meat was then squeezed to release the oil. Next, the oil is collected and filtered. At the end of this procedure, the *Pliek U* oil is produced and labeled as PUO.

A separating funnel was filled with 250 ml of *Pliek U* oil and 300 ml of 96% ethanol. The ethanol fraction was separated into a Baker glass; 300 ml of 96% ethanol was added back into the separating funnel. The ethanol fraction was separated and collected with the previous fraction. The ethanol fraction was then concentrated with a rotary evaporator at 50°C. This procedure gives ethanolic *Pliek U* extract, which is labeled as EPUOE.

### 2.2. Sample Preparation for GC-MS

For GC-MS analysis, 1 gram of PUO and EPUOE extracts were firstly dissolved into *n*-hexane p.a. and vortexed for 2 minutes, respectively. Then, the hexane phase was separated and moved into the derivation tube and then dried with nitrogen bursts. After that 2 ml of 2% NaOH (NaOH in methanol) was added, sealed tightly, and heated at 90°C for 5 minutes, and it was left until cold. 2 ml of BF_3_ in methanol was added, sealed tightly, and reheated for 30 minutes. After being left to cool down, it was extracted with 3 ml *n*-hexane p.a. The *n*-hexane phase (upper phase) was taken for GC-MS analysis.

### 2.3. GC-MS Data Analysis

The peaks of the chromatogram were identified based on MS data analysis to determine the fatty acid content. The percentage of each fatty acid was determined by area percentage (%) of each peak, which later was employed in the determination of predominant fatty acid of each sample.

### 2.4. Docking Procedure

The 3D structure of control and target compounds was downloaded from PubChem database. Meanwhile, the 3D structure of the protein was downloaded from the database of Protein Data Bank (GDP). Then, the natural ligand on the target protein was cut using PyMOL software (https://pymol.en.softonic.com/?ex=DSK-1262.10). After that, the docking process between the control compound and prediction compound of the target protein was carried out using PyRx computer software (https://pyrx.sourceforge.io/downloads).

### 2.5. Visualization of Docking Results

Visualization of docking result was performed by PyMOL and Discovery Studio software (https://www.3dsbiovia.com/products/collaborative-science/biovia-discovery-studio/visualization-download.php).

## 3. Results and Discussion

### 3.1. Fatty Acid Analysis of *Pliek U* Oil by GC-MS

The analyzed data of GC-MS, either for PUO or EPUOE, indicate similar predominant fatty acid content. It is observable that each sample has a different percentage of fatty acids. It can be seen from Table 1 that the highest fatty acid content is in lauric acid (PUO = 30.13% and EPUOE = 36.95%), followed by myristic acid (PUO = 22.25% and EPUOE = 23.55%), palmitic acid (PUO = 15.460% and EPUOE = 12.925%), oleic acid (PUO = 13.476% and EPUOE = 9.980%), stearic acid (PUO = 9.337% and EPUOE = 5.722%), capric acid (3.063% and 4.433), linoleic acid (3.367% and 2.257%), caprylic acid (2.281% and 3.397%), and elaidic acid (0.363% and 0.323%) .

Based on the GC-MS analysis, the predominant fatty acid in *Pliek U* oil in this research was similar to the predominant fatty acids found in the coconut oil reported [20]. The predominant composition of fatty acids in coconut oil per the GC-MS results was lauric acid (47.7%), myristic acid (19.9%), caprylic acid (7.6%), and oleic acid (6.2%). The difference in predominant fatty acid contents was only in palmitic acid, where it was found to be higher in *Pliek U* oil. This difference was probably associated with the geographical factor, where the coconuts were cultivated (Laureles, 2002). Based on these data of fatty acid contents, *Pliek U* oil possibly has a significant potential to be utilized for medical purposes, particularly for atopic dermatitis treatment.

### 3.2. Molecular Docking Results Based on Affinity Binding Value

The common virtual docking of PyRx software was used to dock the 3D structure of ligands (caprylic, capric, lauric, linoleic, myristic, and oleic acids) and the control ligand (palmitic acid) to the target protein (filaggrin). The result of this molecular docking process is the affinity binding value between each ligand to the target protein presented in Table 2.

The binding affinity between fatty acids and filaggrin showed that only oleic acid had a lower binding affinity (−6.1 kcal/mol) compared with the control ligand of palmitic acid (−5.9 kcal/mol). The other ligands showed a greater binding affinity than palmitic acid. The low binding affinity value is associated with the interaction stability between the ligand and the protein. The low binding affinity indicates that the interaction between the ligand and the protein requires low energy. Gibbs's energy theory states that the smaller the energy generated from a bond between the ligand and its receptor, the more stable the bond is [14]. Therefore, oleic acid had the most stable bond to bind filaggrin, in comparison with other fatty acids. In drug design, the stability of interaction between a drug (ligand) and a receptor (target protein) is important to identify the molecular and macromolecular interactions because they determine the therapeutic effects. This information is crucial for the dosage of medicine so that medical treatment is in accordance with what is expected.

### 3.3. Visualization of Fatty Acid and Filaggrin Interaction

Interaction visualization with molecule attachment was conducted to observe the interaction similarity of the control ligand (palmitic acid) and the prediction ligand (caprylic, capric, lauric, linoleic, myristic, and oleic acids) with the target protein (filaggrin). The visualization was carried out with PyMOL software. The obtained information includes the type and the position of the bonding/interaction between the ligand and filaggrin. The optimum interaction was determined based on the interaction similarity between the control ligand with filaggrin and prediction ligand with filaggrin, as well as the number of ligand interaction on protein binding sides. Palmitic acid interaction and filaggrin formed through hydrogen bond and hydrophobic interaction. The hydrogen bond was formed on the THR D71 amino acid residue, while hydrophobic interaction was formed on amino acid residues MET A76, ILE D62, LEU A80, LEU A44, PHE A56, and LEU D75.

#### 3.3.1. Visualization of Capric Acid and Filaggrin Interaction

**Figure 1:**
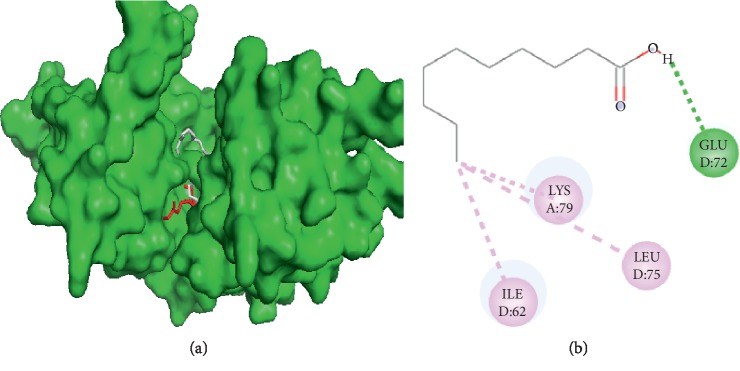
Interaction of capric and palmitic acids with filaggrin. (a) 3D visualization: capric acid (red) and palmitic acid (grey). (b) 2D visualization: amino acid residues in capric acid and palmitic acid.

Capric acid interacts with filaggrin (Figure 1) on the GLU D72 amino acid residue through hydrogen bond and ILE D62, LYS A76, and LEU D75 through hydrophobic interactions. Therefore, capric acid and palmitic acid had two same side interactions to filaggrin, namely, ILE D62 and LEU D75.

#### 3.3.2. Visualization of Caprylic Acid and Filaggrin Interaction

**Figure 2:**
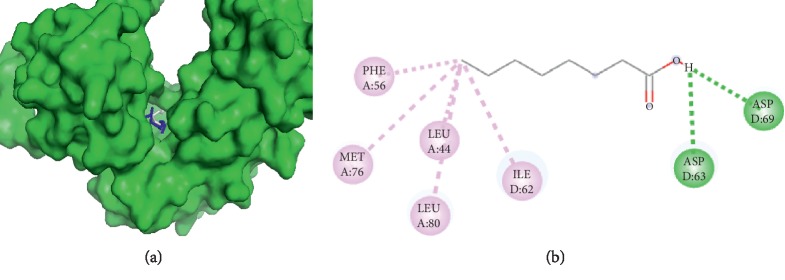
Interaction of caprylic and palmitic acids with filaggrin. (a) 3D visualization: caprylic acid (blue) and palmitic acid (grey). (b) 2D visualization: amino acid residues in caprylic acid and palmitic acid.

The visualization results between caprylic acid and filaggrin (Figure 2) showed the presence of hydrogen bonds and hydrophobic interactions. Hydrogen bonds occur on amino acids ASP D69 and ASP D63, and meanwhile the hydrophobic interaction occur on PHE A56, MET A76, LEU A44, LEU A80, and ILE D62 amino acid residues. Based on the amino acid residues of palmitic acid and caprylic acid, filaggrin is bound on the same sides on the amino acid residues PHE A56, MET A76, LEU A44, and ILE D62.

#### 3.3.3. Visualization of Lauric Acid and Filaggrin Interaction

**Figure 3:**
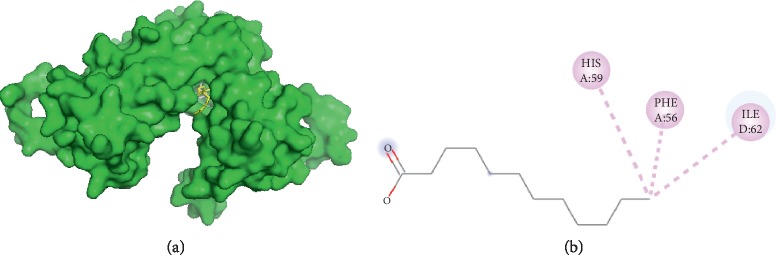
Interaction of lauric and palmitic acids with filaggrin. (a) 3D visualization: lauric acid (yellow) and palmitic acid (grey). (b) 2D visualization: amino acid residues in lauric acid and palmitic acid.

The interaction between lauric acid and filaggrin (Figure 3) only occurs through hydrophobic interactions. The interaction happened on amino acid residues HIS A59, PHE A56, and ILE D62. Therefore, capric acid and palmitic acid had two similar interaction sides to filaggrin, namely, PHE A56 and ILE D62.

#### 3.3.4. Visualization of Linoleic Acid and Filaggrin Interaction

**Figure 4:**
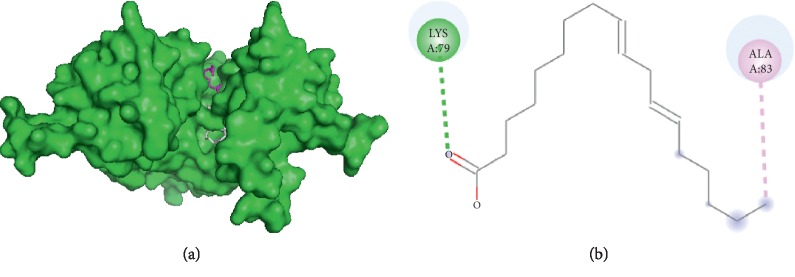
Interaction of linoleic and palmitic acids with filaggrin. (a) 3D visualization: linoleic acid (purple) and palmitic acid (grey). (b) 2D visualization: amino acid residues in linoleic acid and palmitic acid.

Linoleic acid interacts with filaggrin (Figure 4) on amino acid residue LYS A76 through hydrogen bond and amino acid residue ALA A83 through hydrophobic interaction. Therefore, linoleic acid and palmitic acid do not have any same side interaction with filaggrin.

#### 3.3.5. Visualization of Myristic Acid and Filaggrin Interaction

**Figure 5:**
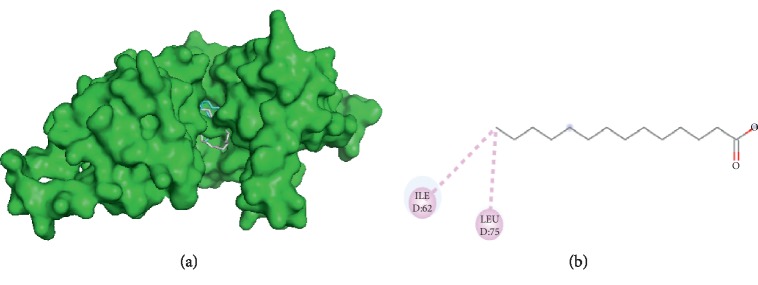
Interaction of myristic and palmitic acids with filaggrin. (a) 3D visualization: myristic acid (light blue) and palmitic acid (grey). (b) 2D visualization: amino acid residues in myristic acid and palmitic acid.

The visualization results between the myristic acid and filaggrin (Figure 5) showed the presence of a mere hydrophobic interaction. The interaction occurred on amino acid residues ILE D62 and LEU D75. Based on the fact, palmitic acid, myristic acid, and palmitic acid bound filaggrin on the same sides of amino acid residues, namely, ILE D62 and LEU D75.

#### 3.3.6. Visualization of Oleic Acid and Filaggrin Interaction

**Figure 6:**
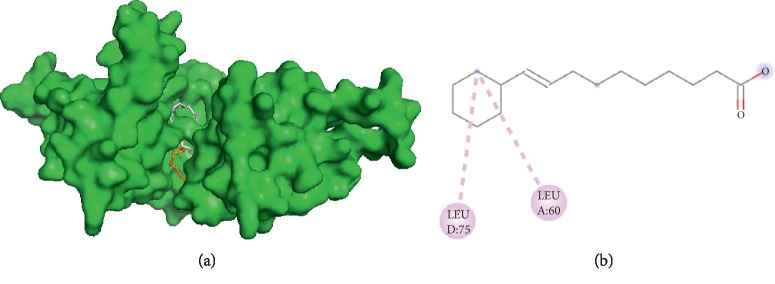
Interaction of oleic and palmitic acids with filaggrin. (a) 3D visualization: oleic acid (orange) and palmitic acid (grey). (b) 2D visualization: amino acid residues in oleic acid and palmitic acid.

The interaction between oleic acid and filaggrin (Figure 6) occurs only through hydrophobic interactions. The interactions were found to be on amino acid residues LEU A60 and LEU D75. Therefore, oleic acid and palmitic acid have one similar interaction side to filaggrin, which is amino acid residue LEU D75.

Visualization of the interaction between fatty acids (predicted ligand) and filaggrin (protein target) reveals that all fatty acids and palmitic acid (control ligand) have the similar binding side to filaggrin, except linoleic acid. Especially for caprylic acid, it has four same binding sides with palmitic acid, which is quantitatively the highest one among the other fatty acids. Based on this result, it can be concluded that caprylic acid has the best structural conformation to bind filaggrin with the best orientation of interaction.

According to the molecular docking analysis, oleic acid gave the most stable interaction with filaggrin, and linoleic acid showed the best fit orientation to bind filaggrin. So, generally, both oleic and linoleic acids have the potential to bind filaggrin well as a drug in atopic dermatitis treatment. Besides having the most stable interaction with filaggrin, oleic and palmitic acids have same binding side with filaggrin. The same binding sides between oleic acid with filaggrin and palmitic acid (control ligand) with filaggrin showed the fit medical interaction between oleic acid and filaggrin. Both the interaction stability and the fit interaction between oleic acid and filaggrin raise the effectiveness of oleic acid as a drug in atopic dermatitis treatment. The bond between oleic acid and filaggrin was formed via hydrophobic interaction on LEU D75. Hydrophobic interactions generated by oleic acid caused the displacement of water molecules so that the entropy value is changed. The interaction between oleic acid and amino acid residues resulted in an increase in the entropy value which drives (catalyzing) the interaction between the protein and ligand. Therefore, hydrophobic interactions have an important role in the ligand and protein binding [21].

In comparison, a placebo-controlled study [22] has been conducted to investigate the effects of seed oil and pulp oil from sea buckthorn (*Hippophae rhamnoides*) in atopic dermatitis patients. The concentration of oleic acid detected is 19% as the main fatty acid in seed oil and 26% in pulp oil. The results showed that there were no changes in triacylglycerol levels, serum total, or specific immunoglobulin E detected. However, Fellermeier [23] who assessed the relationship between intake of selected foods and fatty acids with the prevalence of atopic disease in adults showed that in women, high total fat, palmitoleic acid, and oleic acid intake was positively associated with sensitization. High total fat consumption, high monounsaturated fatty acids, and high oleic acid are positively associated with fever. Thus, the oleate present in this oil is more appropriate for oral use. Ferreri et al. [24] reinforce that radical stress is related to the inflammatory process of atopic disease which is directly related to trans-FA (fatty acids) detected in patients with this treatment. Trans lipid isomers were found in both layers of the skin cell membrane, with a sequential total content of 2.7% and 4.9% of the FA composition. Using an in vitro model, trans lipid isomers were detected by the isomerization of thiyl radicals-catalysts, namely, the appearance of oleic and arachidonic acid isomers. This study shows that oleic acid is appropriate for atopic dermatitis in terms of nonoral aspects of in vivo reaction because it does not cause an immune response (e.g., the appearance of immunoglobulin E).

The epidermis plays an important role in protecting human subjects from exogenous stressors and helps maintain internal fluid and electrolyte homeostasis [25]. Thus, the appropriate epidermal structure will affect the physical, chemical, biochemical, and immune functions of the skin effectively. Filaggrin, one of the epidermal proteins, is very important for the formation of corneocytes and intracellular metabolites, which in turn contribute to maintaining stratum corneum moisture and acidic surface of the skin [26].

Filaggrin is an epidermal protein that is important in controlling the hydration and stratum corneum pH [27]. In patients with a deficiency of filaggrin, it is characterized by the appearance of atopic dermatitis, suggesting that the absence of filaggrin is a key factor in the pathogenesis of this skin condition [28]. Atopic dermatitis is a skin disorder in the form of chronic inflammation characterized by a defect in the epidermal barrier and keratinocyte differentiation. Filaggrin expression is vital because this protein is considered to have a major role in epidermal function [26]. Thus, oleic is a material that is compatible with filaggrin so that it is suitable and has the potential to cure patients/patients with atopic dermatitis who experience impaired skin epidermal function.

## 4. Conclusions

*Pliek U* oil has been successfully analyzed by GC-MS which showed that lauric acid, myristic acid, palmitic acid, and oleic acid were the predominant fatty acid contents. Based on the molecular docking analysis, oleic acid showed the most stable interaction with filaggrin with the lowest binding affinity (−6.1 kcal/mol) compared with others. Oleic and palmitic acids also have one same side binding to filaggrin on amino acid LEU D75. Therefore, oleic acid had the best potential to be used as a drug candidate in atopic dermatitis treatment.

## Figures and Tables

**Table 1:** Fatty acid analysis of PUO and EPUOE by GC-MS.

Component	PUO (%)	EPUOE (%)
Caprylic acid	2.281	3.397
Capric acid	3.063	4.433
Lauric acid	30.130	36.959
Myristic acid	22.255	23.557
Palmitic acid	15.460	12.925
Linoleic acid	3.367	2.257
Oleic acid	13.476	9.980
Elaidic acid	0.363	0.323
Stearic acid	9.337	5.722

**Table 2:** Fatty acid binding affinity of PUO and EPUOE samples.

No	Ligand	Binding affinity (kcal/mol)
1	Capric acid	−5.2
2	Caprylic acid	−4.8
3	Lauric acid	−5.2
4	Linoleic acid	−5.7
5	Myristic acid	−5.6
6	Oleic acid	−6.1
7	Palmitic acid	−5.9

## Data Availability

Data used in this research have not been made available because they are supplementary data and will be used in future work.
